# Vision-based coaching: optimizing resources for leader development

**DOI:** 10.3389/fpsyg.2015.00412

**Published:** 2015-04-15

**Authors:** Angela M. Passarelli

**Affiliations:** Department of Management and Marketing, College of Charleston, Charleston, SC, USA

**Keywords:** vision, leadership, executive coaching, positive emotions

## Abstract

Leaders develop in the direction of their dreams, not in the direction of their deficits. Yet many coaching interactions intended to promote a leader’s development fail to leverage the benefits of the individual’s personal vision. Drawing on intentional change theory, this article postulates that coaching interactions that emphasize a leader’s personal vision (future aspirations and core identity) evoke a psychophysiological state characterized by positive emotions, cognitive openness, and optimal neurobiological functioning for complex goal pursuit. Vision-based coaching, via this psychophysiological state, generates a host of relational and motivational resources critical to the developmental process. These resources include: formation of a positive coaching relationship, expansion of the leader’s identity, increased vitality, activation of learning goals, and a promotion–orientation. Organizational outcomes as well as limitations to vision-based coaching are discussed.

## Introduction

The practice of executive coaching has been widely adopted as a leader development strategy by organizations (Day, 2001; Feldman and Lankau, 2005; Bono et al., 2009). Executive coaching is generally defined as an individualized intervention in which a skilled professional works one-on-one with a leader to identify and achieve his or her personal development objectives (Peterson, 1996; Boyatzis et al., 2006; Coutu and Kauffman, 2009). Although these objectives primarily involve improving effectiveness at work (Feldman and Lankau, 2005), there is evidence that coaching engagements also frequently address non-work topics (Coutu and Kauffman, 2009).

Not surprisingly, the rapid growth of coaching practice has outpaced research (Bennett, 2006). Thus, many executive coaches structure their work by adopting frameworks and models that reflect popular practices in the industry rather than an empirical evidence base (Lowman, 2005). The pressure of a results-oriented business culture has exacerbated the lack of empirical evidence. Together these factors have contributed to the widespread acceptance of heavily assessment-based, goal-centered approaches to executive coaching. Traditionally, these approaches begin with presentation of assessment feedback (e.g., multirater/360-degree feedback, personality assessment data) from which goals are derived and outcomes are measured (Feldman and Lankau, 2005). Although feedback, goal setting, and progress evaluation are valuable components of a coaching process, this paper argues that an emphasis on the leader’s vision for the future cultivates long-term development more effectively than an emphasis on his or her immediate goals.

This paper proposes vision-based coaching as a theory-based alternative to traditional coaching approaches. In contrast to using feedback as the primary intervention strategy, vision-based coaching emphasizes exploration and articulation of an individual’s ideal self as the driver of the developmental process. Grounded in a growing body of research on intentional change theory (ICT; Boyatzis, 2001, 2006, 2008), vision-based coaching holds that emphasizing one’s personal vision evokes a growth-oriented psychophysiological state that gives rise to resources that are crucial to the developmental process. Specifically, vision-based coaching is postulated to improve over traditional approaches by accelerating the formation of positive coaching relationships, facilitating leader identity expansion, increasing vitality or energy for change, activating learning-oriented goals, and fostering a promotion-oriented self-regulatory stance in the person being coached. These motivational resources are proposed to contribute to long-term leader development and positive outcomes at the organizational level. The paper concludes by examining the limitations of vision-based coaching and offering recommendations for future research and practical implementation.

### Connecting Theory to Practice in Vision-Based Coaching

Born out of a practical need to address the short-comings of existing leadership training interventions, executive coaching has evolved based on lessons of experience rather than theoretical grounding. As a result, a plethora of models exist in the practitioner literature, but relatively few have been subjected to rigorous scientific evaluation (for a review, see Grant, 2011). Although scholars have begun to link psychological traditions such as behaviorism, humanism, gestalt, and positive psychology to the coaching process in handbooks and practitioner magazines (e.g., Passmore et al., 2013), theory-based examinations of coaching phenomena are surprisingly absent from peer-reviewed journals. A recent exception is Gregory et al.’s (2011) application of control theory to explain how coaching can enhance behavior regulation via goal monitoring and feedback. The need for theory-based coaching models not only supports grounded practice but also advances the field of coaching through scholarly examination of coaching processes.

A potential shortcoming of coaching models derived from practice is their susceptibility to economic, technological, and socio-cultural influences of the business environment. For example, many coaching engagements begin with multirater (i.e., 360-degree) feedback and identification of short-term objectives (Feldman and Lankau, 2005), presumably in an effort to demonstrate return-on-investment. Additionally, popular coaching models such as GROW (Goals, Reality, Options, Wrap-Up/Way-Forward; Whitmore, 1992; Alexander, 2010) and GAPS analysis (Goals, Abilities, Perceptions, Standards; Peterson, 1996) advocate early identification of goals. These goals are derived through reflective exercises that provide information that is “personally relevant to achieving their goals” (Peterson, 1996, p. 79), such as writing a personal mission statement, values clarification exercises, or career preference assessments. Yet these models put a focus on the client’s present reality, and—if used in isolation—may lead to a process of arriving at goals that circumvents the deep reflective work necessary for organizational leaders to identify their ideal selves. In fact, Jinks and Dexter (2012) suggest that many coaches “…do not spend enough time or use appropriate refinement around facilitating exploration of a broader picture of a client’s preferred future before focusing on specific goals” (p. 103). Focused goals without the context of a long-term vision can result in short-term behavior modification but may lack the emotional commitment required to sustain one’s strivings over an extended period of time. In executive coaching, this is of particular importance because development unfolds over the course of a leader’s career, often requiring months or years to master various leadership capabilities (Lord and Hall, 2005).

**FIGURE 1:**
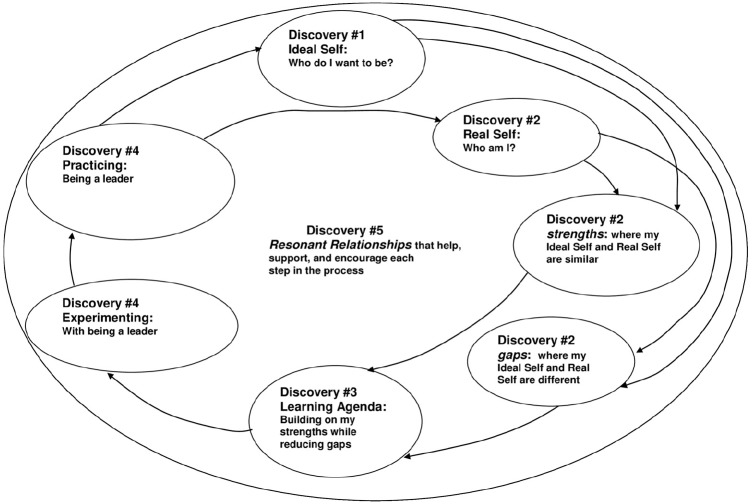
**Intentional change theory.** Reprinted from Boyatzis (2008, p. 304). Copyright 2008 by the American Psychological Association. Reprinted with permission.

Intentional change theory (Boyatzis, 2006, 2008) outlines a developmental process that occurs as leaders create enduring personal change and, hence, provides a foundation for executive coaching. Having evolved from self-directed learning theory (Kolb and Boyatzis, 1970), ICT addresses mechanisms of identity, affect, and physiology that underpin enduring behavior change. Specifically, ICT holds that sustained, desired change occurs in a dynamic, non-linear process punctuated by five discoveries or epiphanies: (1) discovery of the ideal self, (2) assessment of the real self as compared to the ideal self, (3) formulation of a learning agenda, (4) practice and experimentation with new behaviors, and (5) the support of resonant relationships (Figure 1; Boyatzis, 2008). Discovery of the ideal self entails articulating one’s deepest aspirations, hopes, and dreams for the future, as well as positive aspects of one’s core identity. The real self involves examining one’s current strengths and weakness in relation to the ideal self. A learning agenda comprised of broad goals and specific actions is devised in order to bring an individual closer to his or her ideal self. Practice and experimentation is the step by which the learning agenda is implemented and refined. Finally, a set of trusting, growth-fostering relationships supports each discovery.

Fundamental to ICT is the notion that change must be *desired* to endure (Boyatzis, 2008). In situations where individuals’ developmental efforts are in response to external standards, demands, or mandates, the desired end state is typically compliance or approval rather than lasting change. On the other hand, a clear image of one’s ideal self provides a source of motivation and commitment to behave in ways that reduce the discrepancy between one’s current state and the ideal (Higgins, 1987).

Drawing on this tenant of ICT, vision-based coaching advocates for the ideal self to play a central role in the coaching process. In practice this translates to guiding the leader through visioning exercises to explore his or her ideal self as a starting point for the coaching process. Visioning culminates with a detailed articulation of the ideal self, such as a personal vision statement. The personal vision statement then provides an artifact to be referenced and revised throughout other discoveries in the coaching process. A personal vision is distinct from goals in that it is more aspirational, holistic, and distal than goals, which tend to be more instrumental, targeted, and proximal. Goals do play a role in vision-based coaching, particularly in setting a learning agenda. In this way, vision-based coaching is not incompatible with other coaching modalities referenced above. In fact, ICT may provide a macro-structure in which more targeted coaching practices, such as motivational interviewing or cognitive-behavioral techniques, can take place.

### The Role of Vision in Leader Development

The ability to create and convey a compelling vision for the organization is a cornerstone of transformational and charismatic leadership (Conger and Kanungo, 1987, 1998; Bass and Avolio, 1993; Bass, 1998). An organizational vision is typically described as having the qualities of being idealized, future-focused, value-laden, and emotionally arousing (House, 1977; Rafferty and Griffin, 2004; Carton et al., 2014). A vision with these characteristics promotes a sense of shared identity among followers (Conger et al., 2000) and inspires ownership of the vision by clearly outlining how members play a role in the future of the organization (Stam et al., 2010).

The aim of vision-based coaching is not to focus on the organization but on the leader. Although a vision for the organization may be a component of the visioning process, discovering the ideal self necessitates exploring the leader’s broader life context. Research suggests this may be foundational to articulating an organizational vision that is congruent with the leader’s own values and self-image. For example, Shipman et al. (2010) found that self-reflection in late stages of formulating a vision for one’s organization interfered with the ability to forecast or envision outcomes.

Vision-based coaching defines vision as the symbolic representation of one’s ideal self. The ideal self, according to ICT, combines the future-focused nature of Higgins et al. (1994) *ideal self* with present state elements of Roberts et al. (2005) *best self*. Specifically, the ideal self is a possible self that is comprised of one’s desired future (aspirations, dreams, passions, and purpose), core identity (values and individual characteristics), and the emotional driver of hope (Boyatzis and Akrivou, 2006). Greater awareness of the ideal self is accompanied by affirming thoughts, a connection to that which is deeply meaningful, and a sense of optimism and self-efficacy that correspond to an increase in positive emotions (Howard, 2006). The ideal self serves as a catalyst for the change process because it creates a discrepancy between one’s current real self and the self to which one aspires (Higgins, 1987; Oettingen, 1995). It also gives rise to a growth-oriented psychophysiological state (Howard, 2006).

A coach assists clients in refining their personal visions through inquiry designed to evoke hope, mindfulness, compassion, or playfulness as one considers the question, *Who do I want to be?* Questions related to the ideal self encourage clients to reflect on their deepest aspirations and dreams (hope), people who have had a positive impact on their lives (gratitude and compassion), and/or their values and core identity (mindfulness). Ideal self-related questions can also have a spirit of fun and adventure (playfulness). This form of coaching has been referred to as *coaching with compassion* (Boyatzis et al., 2006, 2012).

Vision-based coaching stands in contrast to many traditional models of coaching that emphasize a client’s *real self*, self-knowledge about one’s current level of competence, including both strengths and weaknesses, informed by one’s own assessment and the assessment of others (Taylor, 2006). Coaching that is primarily concerned with exploring the question, *Who am I now?*, tends to be prescriptive, relying on externally defined goals to guide the coaching process. Because this approach engenders short-term compliance rather than lasting change, it is referred to as *coaching for compliance* (Boyatzis et al., 2006, 2012). Although awareness of one’s real self is essential to the change process, ICT argues that focusing on the real self in the absence of any exploration of the ideal self is counterproductive to the aims of coaching for leader development.

### Psychophysiological States as Facilitating Mechanisms for Change

Movement through the intentional change process is propelled by vacillation between two psychophysiological states referred to as positive and negative emotional attractors (PEA, NEA; Howard, 2006; Boyatzis et al., 2015). Broadly speaking, the PEA plays a growth-oriented role in preparing the leader emotionally, cognitively, and physiologically for enacting change. The NEA, on the other hand, plays more of a protective role, signaling threats toward which resources should be allocated. Because the PEA and NEA have qualities that are both beneficial and detrimental to sustaining personal change, ICT holds that the sequencing and salience of the PEA and NEA have a profound effect on coaching effectiveness (Howard, 2006).

When the coaching process engages clients in exercises such as envisioning a desired future, reconnecting with personal values, discovering strengths, and expressing gratitude for supportive relationships, the PEA state is evoked (Boyatzis et al., 2006). The PEA state is associated with the experience of positive emotions, cognitive openness, and a greater influence of the parasympathetic nervous system on autonomic functioning (Boyatzis, 2008). Activated by experiences of hope, compassion, mindfulness, and/or playfulness, the PEA has one of two effects—either calming or energizing (Boyatzis and McKee, 2005; Ayan, 2009). The PEA is often associated with—but not limited to—discovery of one’s ideal self. In the reality of executive coaching, the PEA state and the ideal self have a symbiotic relationship. Focusing on one’s personal vision (ideal self) evokes a PEA state. In return, the PEA state facilitates the salience of the ideal among multiple possible selves. The PEA is a distinguishing feature of vision-based coaching and, hence, will be described in more detail in following sections.

The NEA state is associated with the experience of negative emotions, cognitive impairment, and a greater influence of the sympathetic nervous system on autonomic functioning (Boyatzis, 2008). The NEA activates the human stress response and negative emotions that arise from a focus on current deficits, fears, problems, or by a values misalignment (Boyatzis et al., 2006). The NEA is often invoked by the real or perceived need to comply with social expectations, pressures, and controls—the “ought” self—that suppress one’s ideal self (Higgins et al., 1994; Howard, 2006). Certain situations that arise in the context of executive coaching are known to provoke a stronger NEA response than others. These situations involve the perception of a lack of control, the element of social evaluation, low efficacy or commitment to reaching a goal, and/or anticipation of events involving the previous three characteristics (Dickerson and Kemeny, 2004; Sapolsky, 2004; Boyatzis et al., 2009). The NEA is often associated with—but not limited to—the second discovery in the intentional change process, examining one’s real self and, in particular, a focus on gaps or weaknesses. In the coaching process, the NEA can occur during evaluative processes, such as receiving and interpreting 360° feedback. In its most intense form, coaching to the NEA involves a coach imposing goals that serve the interests of an organization over the interests of the client. For example, the NEA will likely predominate when coaching is conducted with the intention of forcing leaders to change against their will or to comply with organizational mandates.

Having some negativity in the coaching conversation is natural and necessary for development. NEA states are beneficial to the change process when they call “attention to behaviors and events that compromise our effectiveness, threaten our safety, drain our resources, increase our stress, or require us to improve or protect ourselves” and are balanced by recurrent activation with the PEA (Howard, 2006, p. 663). However, highly intense or prolonged periods of NEA trigger individual defense mechanisms and may hinder or halt learning and development. Whereas passive negative emotions, such as sadness, lead to greater information processing than positive emotions, more activating negative emotions, such as anger, may lead to snap decision making and self-defeating behaviors that undermine the change process (Leith and Baumeister, 1996). In addition, negative emotions stemming from concerns of social exclusion have also been found to impair executive functioning, critical thinking, and reasoning (Baumeister et al., 2002). Prolonged periods of NEA not only hurt mental health, they also take a toll on one’s physical health (Boyatzis et al., 2006).

Despite the best efforts of a coach to help a client focus on the positive, individuals tend to be drawn to the negative. This is the result of a well-documented “negativity bias,” a psychological phenomenon by which negative events have a greater impact on individuals than positive events (Baumeister et al., 2001). Vision-based coaching provides a buffer to the bias for negative information by evoking PEA states first and frequently throughout the coaching process.

#### Affective, Cognitive, and Physiological Correlates of the PEA

As the name implies, the PEA has a positive emotional valence. Due to the temporary nature of positive emotions (Fredrickson, 2001), coaches must return frequently to the ideal self throughout a coaching engagement to ensure an overall tone of the PEA. Even fleeting experiences of positive emotions, such as joy, interest, contentment, and love, build an individual’s resources to respond effectively to more negative emotional experiences (Fredrickson, 1998). Positive emotions serve as a buffer to chronic stress, providing support for behavioral, cognitive, and biological coping mechanisms (Fredrickson, 2001). Positive emotions contribute to building social bonds and increase the likelihood of cooperation and reciprocity in the coaching relationship (Barsade and Gibson, 2007). Positive emotions also facilitate persistence in learning to the point of mastery (Fredrickson, 1998; Immordino-Yang and Damasio, 2007).

Positive emotions support the developmental process through their links to cognition. For example, positive affective states increase pattern recognition capability, strengthen memory, and enhance creativity (Isen, 1987; Fredrickson, 1998). Positive emotions also broaden attention (Fredrickson and Branigan, 2005) and improve cognitive flexibility, ostensibly through the release of dopamine in the brain (Ashby et al., 1999).

The psychological components of the PEA state are embodied in its physiological correlates (Cacioppo and Tassinary, 1990). PEA states have been associated with autonomic activity that supports social engagement and recovery from stress (increased parasympathetic activity; Porges, 2003), the release of bonding hormones (oxytocin in women and vasopressin in men; Kemp et al., 2012; McCall and Singer, 2012), and neurological activity in regions of the brain associated with social cognition (the default mode network; Jack et al., 2012, 2013b). Together, these correlates contribute to a positive physiological state Heaphy and Dutton (2008) refer to as “physiological resourcefulness.”

#### Discerning Challenge or Threat States

As mentioned above, the PEA state can be evoked by experiences of mindfulness, compassion, hope, and playfulness (Boyatzis, 2008). These various experiences likely have unique physiological profiles. In fact, examinations of physiological arousal during real-time coaching conversations using the stress hormone cortisol (Howard, 2009) and measures of autonomic activity (Passarelli, 2014) revealed unexpected results. Both of these studies found that discussing one’s vision with a coach for the first time evoked a mild stress response. This phenomenon can be explained by the Biopsychosocial Model of Challenge and Threat (Blascovich, 2007), which holds that individuals’ physiological systems respond to support an assessment of either challenge or threat in active performance situations. A coaching interaction can be considered an active performance situation for both the client and the coach in that it is a goal-relevant activity whereby a certain level of performance is required to maintain wellbeing, and can be perceived in varying degrees as socially evaluative (Tomaka et al., 1993).

Depending on individuals’ assessments of their own resources compared to the demands of the situation, a challenge or threat state will emerge. The “challenge” state occurs when one’s perceived resources are greater than the demands of the situation, resulting in a physiological response that supports optimum performance. This conscious or unconscious appraisal increases sympathetic-adrenomedullary axis (SAM) activity and vasodilation in large skeletal muscles (decreased vascular resistance) with the end product being relatively unchanged blood pressure (Blascovich and Mendes, 2000). Alternatively, when the demands of a situation appear to outweigh an individual’s personal resources, a “threat” state is produced that impairs performance through its associated physiological arousal (Tomaka et al., 1993). Threat is marked by an increase in SAM activity and in the pituitary-adrenal-cortical axis which increases vascular resistance, leading to relatively large increases in blood pressure (Blascovich and Mendes, 2000). According to this theory, both challenged and threatened individuals should exhibit increased cardiac activity during coaching conversations but will differ in vascular resistance. This suggests a reinterpretation of the finding that vision-based coaching conversations did not elicit the physiological element of the PEA state is in order. According to this view, vision-based coaching may elicit a challenge response, which—although physiologically heightened—is an adaptive strategy that allows an individual to mobilize resources necessary to engage in the process of visioning and intentional change. On the other hand, coaching that puts an undue emphasis on the problem and a client’s lack of resources to address it will evoke a threat state, diverting physiological resources from the work of coaching to regulating one’s own emotions and managing the stress of the situation (Mendes et al., 2007). In summary, coaching interactions represent motivated performance situations that elicit physiological challenge or threat states via emphasis on the PEA and NEA, respectively.

Proposition 1: Vision-based coaching activates a PEA state characterized by positive affect, cognitive openness, and a physiological challenge response to a greater degree than coaching interventions that do not include an ideal self-component.

## Developmental Resources

**FIGURE 2:**
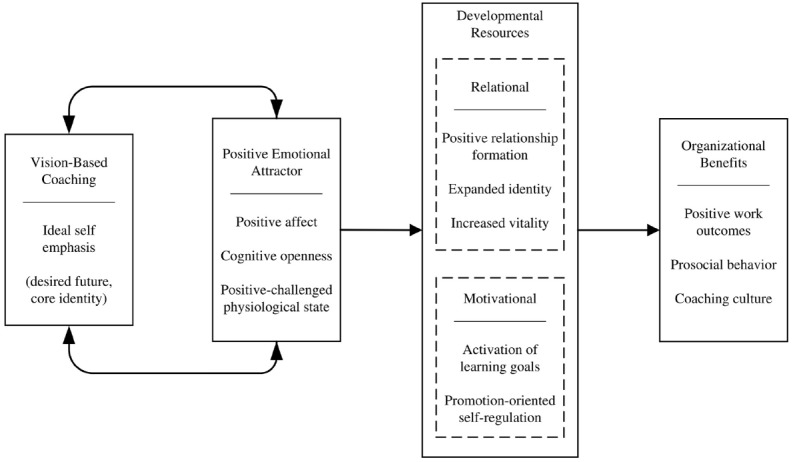
**Conceptual model of resources cultivated through vision-based coaching**.

The PEA state creates the conditions for the emergence of resources that facilitate enduring leader development. For the purposes of this paper, these resources are organized into two categories—relational and motivational (Figure 2). The relational category represents developmental resources stemming from the coaching relationship itself. To the extent that PEA states are shared between the coach and the leader, vision-based coaching offers benefits to both members of the dyad (Boyatzis et al., 2012). However, this paper focuses on the developmental resources of the leader or client, which include facilitation of relationship formation, identity expansion, and enhanced vitality. The motivational category is composed of resources derived from one’s personal vision, including a concern for mastery in the goal setting process (i.e., goal-orientation) and a promotion-oriented self-regulatory focus. Resources in both categories depend on the presence of a PEA state.

### Relational: Vision and Positive Coaching Relationships

The importance of the coaching relationship is not unique to vision-based coaching. Many researchers hold that it is in the context of high-quality relationships that growth and transformation occur (Josselson, 1996; Miller and Stiver, 1997; Dutton, 2003). Similarly, coaching research consistently identifies the quality of the coaching relationship as a key predictor of positive coaching outcomes (Kampa-Kokesch and Anderson, 2001; Bennett, 2006; Gyllensten and Palmer, 2007; Gregory and Levy, 2011). High-quality coaching relationships have been described in a number of ways. In a qualitative study of individuals who went through a workplace coaching process, Gyllensten and Palmer (2007) found positive coaching relationships were established on a foundation of trust and transparency, which promoted psychological safety and active participation in the process. Bluckert (2005) added rapport, support, and challenge as key elements of a successful coaching relationship. According to Gregory and Levy (2010), high-quality employee coaching relationships are evidenced by a genuineness and comfort in the relationship, as well as positive communication and the facilitation of development. When the connection between a coach and a client is marked by these positive qualities, the developmental aims of coaching are achieved more rapidly (Baron and Morin, 2009).

According to ICT, such growth-fostering relationships are the center around which desired, sustained change revolves (Boyatzis, 2008). To be clear, ICT does not suggest that effective coaching relationships require both members of the dyad to be in the PEA state at all times. In fact, recurrent activation of the PEA and NEA is necessary and healthy for the coaching relationship, assuming comparatively more time is spent in the PEA. Acute periods of negativity can be productive in moving a relationship forward because they signal that something in the relationship needs attention which, once resolved, strengthens the relationship. In these cases, the NEA is not a static state but part of the natural evolution of growth-fostering relationships (Jordan and Cooley, 2000). Such relationships have been found in other domains to ease career transitions (Ibarra, 2003), assist in growth and development (Boyatzis et al., 2006; Ragins and Verbos, 2006), enhance and enrich identity (Roberts, 2007), and establish interpersonal trust that facilitates learning from failure (Carmeli and Gittell, 2009; Carmeli et al., 2009). Relationships of this nature also have physiological benefits that contribute to resilience and engagement at work (Heaphy and Dutton, 2008).

#### Positive Relationship Formation

The ability to establish a positive relationship is paramount to executive coaching practice. Early coaching interactions are shaped by relational images, or generalized mental models about what the coaching relationship should entail and how each party should behave (Miller and Stiver, 1997). Relational images built on prior experiences of painful developmental relationships can undermine the formation of a positive coaching relationship (Jordan, 2010). Vision-based coaching offers an alternative for engaging in developmental relationships that can modify existing dysfunctional scripts.

When a coach tunes in to an individual’s ideal self, the best version of themselves they aspire to be, as opposed to their shortcomings, it communicates acceptance and affirmation (Roberts et al., 2005). As the coach demonstrates the empathetic attunement, understanding and sharing in the affective-cognitive experience of the client, the client experiences safety and positive emotional bonding that reinforces the PEA state of both parties (Jordan, 1991; Boyatzis et al., 2006). The PEA state is critical when a new relationship is forming. Positive emotionality is associated with an increased range and depth of self-disclosure (Cunningham, 1988; Vittengl and Holt, 2000). Based on diary studies, those who experience greater positive emotions have more enjoyable social interactions (Berry and Hansen, 1996) and greater friendship closeness (Berry et al., 2000). In a study of college roommates who are getting to know each other in the initial weeks of school, Waugh and Fredrickson (2006) found that those who displayed greater positive emotions also experienced greater self-other overlap (interpersonal closeness) and a more complex understanding of one another. Thus, through the mediating effects of the PEA state, vision-based coaching accelerates the formation of a positive relationship between the coach and the client.

Proposition 2: Vision-based coaching facilitates positive relationship formation.

#### Expanded Identity

Many clients come to coaching with identity-related concerns in their leadership role. These concerns are often more salient during times of career transition. For example, individuals who have been recently promoted to a management position from individual contributor roles may never have viewed themselves as leaders. Others with deep operations management experience may be challenged by a new, more strategic leadership role. Finally, individuals who have recently entered a new organizational culture may be challenged by a personal approach to leading that varies from the dominant leadership style. In all of these cases, vision-based coaching encourages the leader to explore parts of the self that have been ignored or suppressed by social influences. Through a relational process, the clients expand their senses of self and shape their leader identities.

Vision-based coaching strengthens the positive aspects of one’s identity (Roberts, 2007). In addition to drawing out aspects of the self that are aspirational in nature, vision-based coaching helps a leader reflect on current strengths and values as a basis for personal growth. It turns the individual’s attention to examples of personal effectiveness and social information that highlights one’s personal characteristics at his or her best (Roberts et al., 2005). As individuals gain affirmation about these positive components, they begin to expand their self-view to be consistent with others they deem important in their lives, including the coach (Tice and Wallace, 2003). Additionally, vision-based coaching provides a secure relational foundation that facilitates feedback-seeking behavior, which can further develop one’s identity (Kumashiro and Sedikides, 2005).

An expanded sense of self provides a foundation for forming, reformulating, or deepening one’s self-view as a leader. This self-view, or leader identity, is a sub-component of one’s overall identity that is influenced through both intrapersonal and interpersonal processes (DeRue and Ashford, 2010). By eliciting self-reflection related to one’s leader identity, vision-based coaching increases the salience of this aspect of the self thereby releasing positive motivational effects. As leader identity becomes central to one’s sense of self, he or she is more likely to seek out opportunities to develop as a leader (Day and Harrison, 2007). These experiences provide leaders with an increasingly sophisticated set of knowledge structures upon which they can draw to guide future behavior (Lord and Hall, 2005). Furthermore, a clear and integrated leader identity motivates individuals to behave in ways congruent with their identity (Day and Harrison, 2007). The ability to quickly and efficiently access knowledge relevant to leadership challenges translates to increased skill and maturity in one’s leader identity. In a positive feedback loop, this more salient leader identity enhances the self-regulatory capacity to sustain interest in developmental activities over the months and years it takes to develop as a leader (Lord and Hall, 2005).

Proposition 3: Vision-based coaching facilitates leader identity development.

#### Increased Vitality

Verbal discourse about one’s personal vision with a coach releases deep psychic energy (Josselson, 1996; Boyatzis et al., 2006; Fritz et al., 2011). This feeling of being fully alive and energized, referred to as subjective vitality or zest, invigorates clients to take action toward their visions (Miller and Stiver, 1997; Ryan and Frederick, 1997). Vitality can move a client to adopt a new mindset or challenge a deeply held belief, to try a new behavior, to reflect more deeply, or even to make a major life change. Furthermore, increases in subjective vitality predict sustained efforts toward behavior change (Niemiec et al., 2010).

To be certain, increased vitality is a byproduct of sharing one’s vision with a coach. As coaches draw out and encourage clients’ ideal selves, they transmit relational energy which evokes the PEA state and has an uplifting effect on clients (Owens and Baker, 2011). Initial results from a study examining the neurological correlates of vision-based coaching substantiate the importance of the relational interplay between a coach and client. Specifically, the study found that the ventromedial prefrontal cortex (VMPFC), a region of the brain associated with social support (Eisenberger et al., 2011) and deriving affective meaning from cognitive information (Roy et al., 2012), was activated in participants who talked to a coach about their ideal selves (Jack et al., 2013a). This region became more active as a function of the number of coaching sessions an individual had with his or her coach (i.e., the more PEA sessions a participant had with a coach, the greater the neurological response in the VMPFC). More importantly, this region was not activated among participants who were instructed to type their answers to vision-based questions into a computer rather than interacting directly with a coach. This underscores how essential the *relationship* is to empowering clients through the energizing benefits of vision-based coaching.

Proposition 4: Vision-based coaching enhances subjective vitality.

### Motivational: Vision and Regulation of Goal-Directed Behavior

Although discovery of one’s ideal self in the context of a resonant coaching relationship energizes positive action, creating and executing an agenda for intentional development is an integral part of vision-based coaching (Boyatzis, 2008). Thus, vision-based coaching moves beyond the articulation of one’s ideal self to planning, acting, and monitoring progress toward vision-relevant goals. In the context of leader development, this is a complex task that occurs over an extended period of time (Lord and Hall, 2005). Accordingly, coaches must attend to the motivational resources that enable sustained behavior change even if the coaching engagement is relatively short.

There is evidence that both vision-based and traditional coaching approaches assist clients in setting and pursing goals to a greater extent than they would accomplish alone (Howard, 2009; Grant et al., 2010; Grant, 2012). For example, Grant (2012) demonstrated that coaching questions that focused on a problem and those that focused on a solution both helped participants feel they were moving closer to their goals. However, in addition to emotional and efficacy-related benefits of the solution-based questions, participants in this condition reported feeling significantly closer to achieving their goal and developed more action strategies for attaining it. Further, Howard (2009) studied the effects of emotional attractors in the context of live coaching sessions. Conversations characterized by both the PEA and the NEA facilitated goal setting, and goal setting was associated with negative affect in both conditions. However, negativity associated with goal setting in the PEA condition was significantly less severe than in the NEA condition. These studies suggest that the distinction between vision-based coaching and other approaches is embedded in the *nature* of the goals clients set and the degree to which these differences affect striving toward one’s goals. Specifically, vision-based coaching is posited to support complex goal pursuit by bringing aspirational goals to the forefront of clients’ concerns and optimizing individual motivational orientation.

#### Activation of Learning Goals

Vision-based coaching helps clients formulate goals that are consistent with the long-term demands of learning and development. This occurs as the client adopts a mindset focused on enhancing one’s abilities, thereby activating development-relevant goals and bringing them into focus (Dweck and Leggett, 1988). Even when the coach is physically absent, the psychological presence of a relationship partner—simply *thinking* about the coaching in his or her absence—can activate goals that are congruent with that relationship (Fitzsimons and Bargh, 2003). Thus, vision-based coaching can have a lasting effect on how clients orient toward their goals.

Research on goal orientation suggests that qualitative differences in the nature of goals are associated with differences in goal pursuit and attainment (Seijts et al., 2004). Performance-oriented goals focus on a short-term outcome by which one’s ability can be demonstrated to others. These are most effective when the task is routine or straightforward and an individual already has the ability to perform effectively. On the other hand, learning-oriented goals focus on the process of knowledge acquisition and skill development and are most effective when the task is novel or requires creativity, discovery, or mastery (Seijts et al., 2004; Seijts and Latham, 2005).

Furthermore, Seijts and Latham (2005) suggest that setting a performance goal early in the change process may actually be detrimental because it deters cognitive resources from exploration and discovery necessary for learning. A study by van Hooft and Noordzij (2009) supported this assertion. They found that job seekers who took a learning approach demonstrated greater search intentions, more search behavior, and had higher re-employment probabilities than those with a performance orientation. Additionally, the motivational benefits of learning oriented goals may be most vital in helping individuals persist through adversity (Dweck, 2002; Grant and Dweck, 2003; Blackwell et al., 2007). Thus, the activation of learning-oriented goals serves as a motivational resource for leader development.

Proposition 5: Goals that rise from vision-based coaching will evidence a stronger learning orientation than performance orientation.

#### Self-Regulatory Focus

By emphasizing one’s dreams and aspirations, vision-based coaching not only activates learning goals, but also facilitates sustained goal pursuit by evoking a promotion-focus to self-regulated behavior. Promotion is one of two motivational orientations proposed by Higgins (1997, 1998) in his theory of regulatory focus. The other is a prevention focus. Promotion-focused individuals are motivated to achieve reward, whereas prevention-focused individuals are motivated to avoid negative outcomes. Self-regulatory focus has both trait and state properties, meaning it is a stable feature of one’s personality yet can also be shaped by meaningful coaching interactions (Förster and Higgins, 2005). Thus, vision-based coaching can elicit a promotion orientation because of its focus on the ideal, as well as a distal time orientation (Pennington and Roese, 2003).

When externally primed with a promotion focus, individuals represent goals as aspirations and ideals, utilize approach strategies of goal pursuit that are eager and exploratory in nature, and are concerned with self-fulfillment and growth. Conversely, those in a prevention focus represent goals as responsibilities and duties, utilize avoidance strategies of goal pursuit that are vigilant and cautious, and are concerned with security and safety (Förster and Higgins, 2005).

Promotion and prevention orientations correlate with perceptual processing style. Promotion orientation is associated with a more abstract, global processing, whereas prevention orientation is associated with concrete, local processing (Förster and Higgins, 2005). This is consistent with findings from a recent fMRI study in which PEA-based coaching was found to activate neural circuits associated with higher visual processing and global attention—the same network that is associated with promotion-oriented motivation. Accordingly, NEA-based coaching and local visual attention were found to share an overlapping network associated with a prevention orientation (Passarelli et al., 2013). Thus, the ability to see the forest rather than the trees in a promotion-oriented state arises from our neurological structure.

Promotion–orientation is not always more valuable than a prevention orientation. For instance, a prevention focus is associated with greater performance when undertaking a specialized task requiring careful attention (Förster et al., 2003), when action must be quickly initiated (Freitas et al., 2012), or when the client believes that human intelligence is fixed (Sue-Chan et al., 2012). However, a prevention orientation may undermine developmental efforts where change is required. Research by Zhang et al. (2014) documents a “prevention-repetition effect” in which individuals with either a chronic or experimentally induced prevention focus were more likely to repeat dysfunctional behaviors in an effort to maintain the status quo. Given a tendency to repeat past performance, it maybe exceedingly difficult for prevention-oriented individuals to overcome dysfunctional patterns. Readjusting one’s focus to a promotion orientation may open them to considering alternatives and selecting better behaviors, thereby breaking the cycle of dysfunction. Finally, a promotion orientation is more effective in regulating behavior with regard to complex and ambiguous tasks (Förster et al., 2003). In fact, Sue-Chan et al. (2012) found that promotion-oriented coaching led to greater problem-solving performance than prevention-oriented coaching in studies conducted in both laboratory and field settings. Thus, a promotion orientation gleaned from vision-based coaching will assist in regulating goal-directed behavior.

Proposition 6: Vision-based coaching inspires state-level promotion orientation.

## Organizational Benefits

Although the discussion of vision-based coaching for leader development may appear to have predominantly individual-level benefits, there are also benefits to the organization. Vision-based coaching enhances work outcomes, inspires prosocial behavior, and “spreads” a culture of development.

Boyatzis et al. (2006) hold that vision-based coaching serves as a source of renewal for both the client and the coach. Shared PEA states replenish the psychological and physiological resources necessary to be engaged at work (Loehr and Schwartz, 2003; Boyatzis et al., 2009). Vision-based coaching reinvigorates leaders’ passion for their work and encourages them to express their ideal selves in work tasks and relationships (Boyatzis et al., 2014). Increasing evidence suggests this results in positive work outcomes. For example, a recent study found that vision-based coaching engendered greater work engagement and career satisfaction among financial service executives (Van Oosten, 2013). Similarly, Cable et al. (2013) found that organizational socialization practices that emphasize a newcomer’s ideal self, coupled with the perception that he or she can act authentically, resulted in greater retention, higher quality work, greater engagement and job satisfaction, and more positive work attitudes.

Vision-based coaching often elicits a heightened desire to help others through one’s work or actions (Passarelli, 2014). This desire for enhanced social connections is a common outcome of growth-fostering relationships (Miller and Stiver, 1997). In the short-term, positive affect and a desire to reciprocate may result in leaders taking action that “pays it forward” by engaging in vision-based interactions with colleagues or family members (Barsade and Gibson, 2007). Furthermore, the salience of prosocial values in one’s ideal self may strengthen the likelihood of this discretionary helping behavior (Grant and Dutton, 2012). As leaders themselves experiment with vision-based coaching techniques in their network, a social contagion effect occurs. The PEA state evoked through vision-based coaching spreads through the dynamics of emotional contagion, the tendency to experience and express the emotions of a relationship partner (Hatfield et al., 1994). This transfer of emotions occurs through an unconscious process in which individuals perceive and mimic each other’s emotional cues, such as facial expressions, language, and movement (Cattaneo and Rizzolatti, 2009; Iacoboni, 2009), and is particularly powerful among those who share a sense of interpersonal closeness (Cwir et al., 2011). As the nature of leaders’ conversations change, the culture of the organization will become more developmental.

Proposition 7: Vision-based coaching is positively associated with work outcomes, prosocial behavior, and a shift toward a culture of development.

## Limitations of Visioning

Vision-based coaching advocates for the clarification of one’s ideal self as a starting point for the coaching process and as an anchor for other discoveries involved in intentional change. Although vision gives rise to valuable developmental resources, we recognize that a vision alone is likely insufficient to facilitate behavior change. Rather, a clear vision of one’s ideal self provides a basis for other mental processes, such as mental contrasting and process forecasting, that are germane to subsequent discoveries in ICT and essential for successful goal pursuit. Additionally, visioning “gone wrong” can be counterproductive to the aims of coaching.

### Mental Contrasting

Kappes and Oettingen (2011) warn that idealized images of the future do not take into account the arduous path to attaining that future state, resulting in poorer performance than being in touch with reality. In fact, they suggest that whereas envisioning a desired future has motivational benefits related to increased positive affect, it can erroneously satisfy this need thereby decreasing effort (Oettingen, 1995). By their account, a more effective way to motivate goal-related behavior is through the process of mental contrasting, comparing one’s desired future to current obstacles that might stand in the way. The contrast between the ideal and real becomes the source of motivational energy and commitment to one’s goals (Oettingen et al., 2009).

In a series of studies, participants were asked to (1) imagine a desired future, (2) imagine obstacles and challenges in their current situations that stand in the way of the desired future, or (3) mentally contrast the previous two conditions. Consistently, participants in the mental contrast condition put forth more effort and performed better on the goal-relevant tasks (Oettingen et al., 2000, 2001), which underscores the importance of contrasting the ideal self with the real self in coaching. In addition, mental contrasting calibrates goal commitment with expectancy, such that goal commitment increases when expectations of success are high and *vice versa* (Oettingen et al., 2001). To the extent that vision, via its PEA correlates, buffers the natural proclivity toward negative information in real-self concerns, it may reduce the likelihood that perceived obstacles will erroneously diminish one’s expectations of success thereby leading to increased goal commitment. Finally, mental contrasting research suggests that the connective tissue between expectations of success and goal commitment is physiological and psychological activation or energization (Oettingen et al., 2009). Here again, the vitality-enhancing effects of the vision-based coaching may amplify the energizing effects of mental contrasting.

### Mental Simulation

Research in the areas of sports psychology and addiction suggest further limitations on the relationship between vision and self-regulation. Similar to mental contrasting, research on mental simulation suggests that mentally envisioning a desired end state is insufficient for regulating behavior toward that outcome. Mental simulation differs from mental contrasting in that it posits one must envision the steps necessary to attain a goal rather than contrast the ideal to current reality. Thus, mental simulation or process-based visioning, involves both the ideal end state and the steps necessary to achieve it (Taylor et al., 1998). Mental simulation may contain both real and hypothetical events and is typically constrained by what is plausible (Taylor and Schneider, 1989).

Mental simulation improves self-regulatory capacity by increasing the extent to which an individual believes his or her goal will be achieved (Koehler, 1991). Mental simulation also allows individuals to evaluate multiple solutions to a problem in an environment that approximates the causal chain of events in social reality. It bolsters coping skills by allowing leaders to anticipate and mentally play out their response to high-risk situations. Finally, mental simulation is second only to physical practice in enhancing action readiness (Taylor et al., 1998). Accordingly, process-based visioning, as compared to ideal-only visioning or no visioning, has been linked to superior performance (Pham and Taylor, 1999), planning and problem solving in pursuit of a goal (Taylor et al., 1998), use of active coping strategies in stressful life events (Rivkin and Taylor, 1999), and reduced stress in physical performance situations (Weigert Coelho et al., 2014).

In terms of intentional change, mental simulation may be particularly important as a predecessor to the discovery of practice and experimentation because it allows for mental rehearsal of contextualized behavior. For example, a leader who ideally views him or herself as a charismatic orator might prepare for the next company-wide meeting by playing out how the audience would react to various ways of delivering a message.

### Vision Dysfunction

Vision can interfere with leader development in certain situations. First, escape fantasies not grounded by a clear sense of reality can thwart self-regulatory efforts at development (Oettingen, 1995). Second, the psychophysiological state associated with ideal self-visioning can create openness that is too unfocused or scattered to be usefully directed (Boyatzis, 2013). Third, visioning that takes the form of rumination on painful past or anticipated future experiences can be detrimental to those suffering from mental health disorders, such as depression or post-traumatic stress disorder (Horowitz, 1976).

In addition, it may be counterproductive for a coach to use vision-based techniques when an individual exhibits extreme resistance to exploring his or her ideal self. Excessive emphasis on the ideal self in this situation could violate a leader’s social expectations for the conversation, thus resulting in strain that depletes developmental resources (Fitzsimons and Finkel, 2010). In these situations, it is recommended to use a different approach to foster a PEA state (e.g., discussing important relationships for which one is grateful or values one holds dear) as a “warm up” period to discussing one’s ideal self.

## Conclusion

Organizations invest significant resources in leadership development (Avolio and Hannah, 2008). Ironically, some of these practices may actually deplete the human resources they are designed to augment. Many coaching interactions intended to develop leadership capability are inherently deficit-based, beginning with multisource feedback that triggers a leader’s real self and ensuing NEA state, rather than his or her ideal self and a PEA state. These interactions fail to leverage the transformational power of one’s personal vision, potentially resulting in sporadic or short-term change.

It should be noted that empirical research to date has largely focused on documenting the outcomes of executive coaching—a necessary step for a profession attempting to gain legitimacy. Evidence from these studies suggests that coaching increases leaders’ self-efficacy (Baron and Morin, 2009; Ladegard and Gjerde, 2014); increases satisfaction and commitment and decreases turn-over intentions (Luthans and Peterson, 2003); fosters stronger relationships and personal development, and facilitates work-family integration (Wasylyshyn, 2003). Yet, little empirical evidence demonstrates *how* these outcomes are achieved (Gregory et al., 2008; Segers et al., 2011). That is, the field needs theoretical models for the process by which these outcomes are attained in order to advance beyond outcomes research. The lack of theoretical models has stymied this progress.

Vision-based coaching has been proposed as a theory-driven approach to coaching. The propositions outlined in this paper are intended as a basis for continued empirical research on ICT and the dynamics of executive coaching. This research agenda includes testing the efficacy of vision-based coaching as compared to other approaches, as well as exploring how various approaches might be optimally combined (e.g., modifying the GROW model to include an ideal self-component). The assertion that vision-based coaching leads to “sustained” change requires longitudinal research designs that extend not only the duration of the coaching engagement but also months or years afterward. This research should examine the strength of the proposed relationships over time. Additionally, if support for these propositions is established, boundary conditions must be identified. For example, if vision-based coaching is in fact found to elicit promotion-oriented motivational states, how long does this effect last? Or, how do individual differences moderate the proposed relationships?

The propositions outlined here also have implications for how organizations approach leadership development. First, organizations are called to embed the ideal self in their leadership development initiatives such that participants have an opportunity to consider their vision early and reconnect with it regularly throughout the intervention. Accordingly, this requires reconsideration of the timing of multisource feedback, a key component of many leadership development processes (Day et al., 2014). Second, the propositions included here underscore the importance of frequent experiences of the PEA. This suggests that leader developers and leaders themselves utilize strategies that inspire positive emotions through experiences of hope, mindfulness, compassion, and playfulness. Finally, coach-training programs should include the theoretical basis for practice and, in this case, cover techniques for helping leaders discover their ideal selves.

In summary, vision-based coaching holds that a clear and comprehensive personal vision mobilizes developmental resources through activation of a positive psychophysiological state that optimizes affective, cognitive, and neurobiological functioning for development. These resources fuel ongoing developmental efforts that endure the test of time, benefiting both the leaders being coached and their organizations.

### Conflict of Interest Statement

The author declares that the research was conducted in the absence of any commercial or financial relationships that could be construed as a potential conflict of interest.
